# Atomic Layer Deposition of Mixed-Layered Aurivillius Phase on TiO_2_ Nanotubes: Synthesis, Characterization and Photoelectrocatalytic Properties

**DOI:** 10.3390/nano10112183

**Published:** 2020-11-02

**Authors:** Farid Orudzhev, Shikhgasan Ramazanov, Dinara Sobola, Abdulgalim Isaev, Chuanyi Wang, Asiyat Magomedova, Makhmud Kadiev, Kasinathan Kaviyarasu

**Affiliations:** 1 Department of Inorganic Chemistry and Chemical Ecology, Dagestan State University, st. M. Gadjieva 43-a, Dagestan Republic, 367015 Makhachkala, Russia; ramazanv@mail.ru (S.R.); sobola@vutbr.cz (D.S.); abdul-77@yandex.ru (A.I.); asiyat_magomedova1996@mail.ru (A.M.); k.mahmud@yandex.ru (M.K.); 2 Department of Physics, Faculty of Electrical Engineering and Communication, Brno University of Technology, Technická 2848/8, 61600 Brno, Czech Republic; 3 Central European Institute of Technology BUT, Purkyňova 123, 61200 Brno, Czech Republic; 4 School of Environmental Science and Engineering, Shaanxi University of Science & Technology, Xi’an 710021, China; wangchuanyi@sust.edu.cn; 5 UNESCO-UNISA Africa Chair in Nanoscience’s/Nanotechnology Laboratories, College of Graduate Studies, University of South Africa (UNISA), Muckleneuk Ridge, P.O. Box 392, Pretoria 0003, South Africa; Kasinathankaviyarasu@gmail.com; 6 Nanosciences African Network (NANOAFNET), Materials Research Group (MRG), iThemba LABS-National Research Foundation (NRF), 1 Old Faure Road, 7129, P.O. Box 722, Somerset West 8000, South Africa

**Keywords:** atomic layer deposition, photocatalysis, photoelectrocatalysis, TiO_2_ nanotubes, Aurivillius phase, layered perovskite

## Abstract

For the first time, one-dimensional phase-modulated structures consisting of two different layered Aurivillius phases with alternating five and six perovskite-like layers were obtained by atomic layer deposition (ALD) on the surface of TiO_2_ nanotubes (Nt). It was shown that the use of vertically oriented TiO_2_ Nt as the substrate and the ALD technology of a two-layer Bi_2_O_3_-FeO_x_ sandwich-structure make it possible to obtain a layered structure due to self-organization during annealing. A detailed study by scanning electron microscopy (SEM) and transmission electron microscopy (TEM) showed that the coating is conformal. Raman spectroscopic analysis indicated the structure of the layered Aurivillius phases. Transient photocurrent responses under Ultraviolet–Visible (UV-Vis) light irradiation show that the ALD coating benefits the efficiency of photon excitation of electrons. The results of the photoelectrocatalytic experiments (PEC) with methyl orange degradation as a model demonstrate the significant potential of the synthesized structure as a photocatalyst. Photoluminescent measurement showed a decrease in the probability of recombination of photogenerated electron–hole pairs for ALD-coated TiO_2_ Nt, which demonstrates the high potential of these structures for use in photocatalytic and photoelectrochemical applications.

## 1. Introduction

It is known that the Aurivillius phases of the Bi_4_Ti_3_O_12_-BiFeO_3_ (BFTO) system combine ferroelectric, semiconductor and ferromagnetic properties and that they are potentially attractive for a large number of applications [1,2]. These oxides have a layered perovskite structure where fluorite-like layers (Bi_2_O_2_)^2+^ alternate with perovskite-like layers (Bi_n+1_Fe_n−3_Ti_3_O_3n+1_)^2−^. The index n indicates the number of perovskite-like layers in one block and can be an integer or a fractional value [3]. Fractional values of n correspond to multilayer structures that contain alternating perovskite-like blocks of different layers and appear due to the fact that the Aurivillius phase system is inclined to structural disorder [4,5]. The compounds of the Aurivillius phases have been mostly studied as bulk crystals and ceramics [6,7] and there are only few reports on the synthesis and study of thin films [4]. Preparation of multicomponent oxides by the atomic layer deposition (ALD) method is a difficult task because the search for optimal growth parameters is time-consuming and requires a large number of experiments and resources. Bismuth-containing oxides with layered structures, especially representatives of the Aurivillius family, have attracted increasing attention in recent years as visible-light-active photocatalysts [8,9,10]. The presence of Bi increases the probability of absorption of visible light, shifting the edge of the valence band (VB) due to the hybridization of Bi 6s with the O 2p orbital and contributes to the high mobility of photogenerated holes [10].

The highly oriented arrays of TiO_2_ nanotubes (Nt) have long demonstrated excellent photovoltaic and photocatalytic properties due to their unique morphology, high specific surface area and unidirectional charge transfer, resulting in small losses at grain boundaries due to recombination [11]. A recent review article [12] examined, in detail, the works in which ALD technology was used for the functionalization of TiO_2_ Nt with other materials to improve their physicochemical properties, such as light absorption, separation of photogenerated electron-hole pairs, photocatalytic and photoelectrocatalytic properties, chemical, thermal and mechanical stability and others.

In this work, for the first time, we report on ALD synthesis of the mixed-layer structure of Aurivillius on a substrate of TiO_2_ Nt, using them as an alternative to ALD cycles associated with titanium-containing precursors. Thus, we simplified the ALD technology of multicomponent oxide systems and obtained a structure with a more advanced surface. This approach provides opportunities for new applications in relevant fields, such as photocatalytic decomposition of pollutants and photoelectrochemical water splitting.

## 2. Materials and Methods

### 2.1. Synthesis Procedure

The film was grown in an ALDCERAM ML-200 (ALDCERAM, LLC, Boulder, CO, USA) atomic layer deposition reactor. The substrate used was TiO_2_ Nt on Ti foil, prepared as described in the work [13]. The details of the synthesis procedure were described by us in our previous work [14,15], where the Bi-Fe-O phase was synthesized on the surface of highly oriented pyrolytic graphite (HOPG). In this work, the other number of cycles was used at the ALD process (Bi_2_O_3_ (90 cycles)-FeO_x_ (90 cycles)). The TiO_2_/Ti structure was used as the substrate and the samples were annealed at 660 °C in air for one hour. The source of bismuth was Bi(mmp)_3_ precursor (its chemical formula is C_15_H_33_O_6_Bi) (Sigma-Aldrich, Schnelldorf, Germany). The ferrocene-Fe(Cp)_2_ was used as an iron-containing organometallic precursor (its chemical formula is Fe(C_5_H_5_)_2_) (Sigma-Aldrich, Schnelldorf, Germany).

### 2.2. Characterizations

Characterization of the obtained heterostructures was performed using scanning electron microscopy (SEM) with the scanning electron microscope Magellan (Thermo Fisher Scientific, Hillsboro, OR, USA) and transmission electron microscopy (TEM) with TITAN 80-300 (FEI, Fremont, CA, USA). X-ray diffraction (XRD) studies were done using an Empyrean PANalytical X-ray diffractometer (Almelo, Netherlands) in the radiation of a copper anode with a nickel filter, with radiation wavelength λ(Cu_Kα_) = 0.154051 nm. Data processing was performed using the High Score Plus application program, included in the instrument software, and the diffraction database ICSD (PDF-2). Additional study of the surface composition was carried out by an AXIS SupraTM X-ray photoelectron spectrometer (XPS) (Kratos Analytical Ltd., Manchester, UK). The data were processed by CasaXPS v.2.3.23 software (Casa Software Ltd., Wilmslow, UK). Raman spectra were examined by a Laser Raman 3D scanning confocal microscope (Ntegra Spectra, Moscow, Russia) using a green laser (532 nm) with a spot size of 1 µm and a resolution of 0.5 cm^−1^. Photoluminescence spectra were recorded by a Lyumakhrom Fluorat-02 panorama spectrofluorometer (St. Petersburg, Russia) with a 320-nm wavelength of exciting radiation.

Diffuse reflection spectra were measured by a Shimadzu UV-3600 (Kyoto, Japan) spectrophotometer with an LISR-3100 integrating sphere in the coordinates F(R) = f (λ, nm), where F(R) is the Kubelka–Munk function (denoted in the text as α). We used the method of the Tauc curve plot to determine the band gap by conversion of the optical absorption spectra using the absorption coefficient (α) and photon energy (hν), calculated considering the wavelengths: hν = 1240/λ. The band gap energy was determined by extrapolation of the linear area of the dependence of (αhν) on (hν) to the zero value of the absorption coefficient, where n is equal ½ in the case of indirect and n is equal 2 in the case of direct inter-band transition. Urbach energy values (EU) were calculated by inverse slope of the dependence of lnα on hν.

### 2.3. Photoelectrochemical Measurement

Transient photocurrent responses under Ultraviolet–Visible (UV-Vis) light irradiation were studied by the potentiostat-galvanostat IPC-Pro MF (St. Petersburg, Russia). A saturated silver chloride Ag-AgCl electrode was used as a reference electrode. Platinum electrode was used as the counter electrode. The synthesized samples of pure and ALD-coated TiO_2_ Nt were the working electrodes. The area of the working electrode was 0.25 cm^2^. The distance between the electrodes was 2 cm. During the photoelectrocatalytic experiments (PEC), a solution of freshly prepared methyl orange (MO) dye (30 mL of 5 mg/L concentration) with 0.1 M Na_2_SO_4_ was irradiated by UV-Vis light in quartz cell. The experiment was carried out without a voltage offset. The light source was a 250 W discharge mercury vapor lamp with a preliminary removed phosphor layer and a 365 nm main wavelength. The emission spectrum is given in Figure S1. To study the kinetics of the PEC process, the absorption spectra were measured every 10 min on a Specord 210 plus spectrophotometer (Analytik Jena, Jena, Germany) with a 1 nm step and 10 nm/s speed. After this, the solution was returned to the cell and the process continued. 

## 3. Results

**Figure 1:**
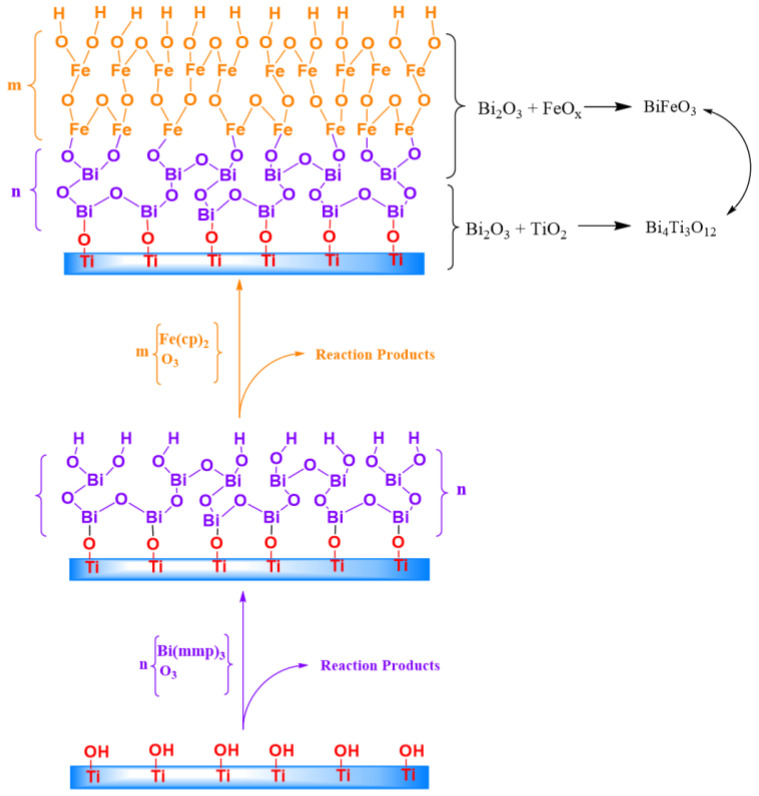
Schematic process of atomic layer deposition (ALD) Aurivillius phase formation.

The intense volatilization of bismuth is one of the fundamental problems in the synthesis of bismuth-containing ternary compositions [16]. Therefore, we decided to use the technology for producing a two-layer sandwich structure from Bi_2_O_3_ (90 cycles)-FeO_x_ (90 cycles), in contrast to the previously proposed homogenous mix technology [17]. Considering the problem of volatilization, we synthesized the first layer of crystallized Bi_2_O_3_. As has been previously shown [18], the alpha phase of bismuth oxide crystallizes already during the growth at 250 °C. The region of Bi_2_O_3_/TiO_2_ is formed at the interface with nanotubes. According to our assumption, the next deposited FeO_x_ layer should limit the volatilization of bismuth during further annealing, since the chemical reaction will occur at active bismuth diffusion through the FeO_x_ layer. Figure 1 shows a scheme of the processes during ALD and subsequent annealing of the sample. 

The compounds of the Aurivillius phases are formed in the Bi_2_O_3_-TiO_2_ composition at n(Bi)/n(Ti) > 1 [19]. The processes of the compounds’ formation at the phase boundary between Bi_2_O_3_ and TiO_2_ in the TiO_2_-Bi_2_O_3_-TiO_2_ sandwich composition were studied by Lu et al. [20]. The authors concluded that first, a stable compound, Bi_4_Ti_3_O_12_, is formed in the Bi_2_O_3_ layer due to diffusion of titanium. In addition, the annealing temperature of 660 °C is close to the melting point of the phase to γ-Bi_2_O_3_ (T_m_ ≈ 550–630 °C). It causes significant increasing in the mass-transfer rate and initiation of chemical reactions, which also contributes to reaction 1.

The intensive mass transfer of bismuth oxide along the surface of the growing film during annealing of the sample leads to a solid-phase reaction in the near-surface region:

3TiO_2_ + 2Bi_2_O_3_ = Bi_4_Ti_3_O_12_ (1)

Due to mass-transfer, the reaction in the Bi_2_O_3_-FeO_x_ region could be described by Equation 2:

2FeO_x_ + Bi_2_O_3_ = 2BiFeO_3_ (2)

**Figure 2:**
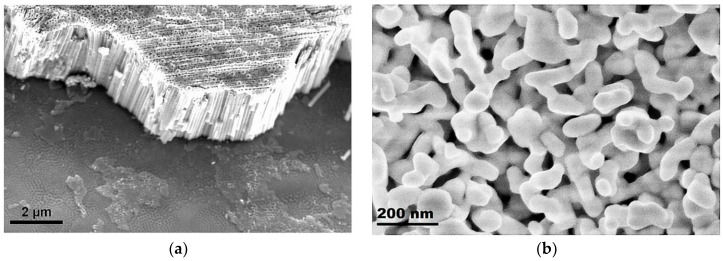
Scanning electron microscopy (SEM) images of (**a**) pure and (**b**) ALD-coated TiO_2_ nanotubes (Nt) on Ti foil.

During annealing, the interaction of two phases, BiFeO_3_-Bi_4_Ti_3_O_12_, as shown in Figure 1, leads to the formation of the layered structure of the Aurivillius phase. The number of layers in this structure is due to the presence of BiFeO_3_ in the perovskite-like block [2]. Figure 2 shows SEM images of the surface of TiO_2_ Nt before and after the deposition process.

**Figure 3:**
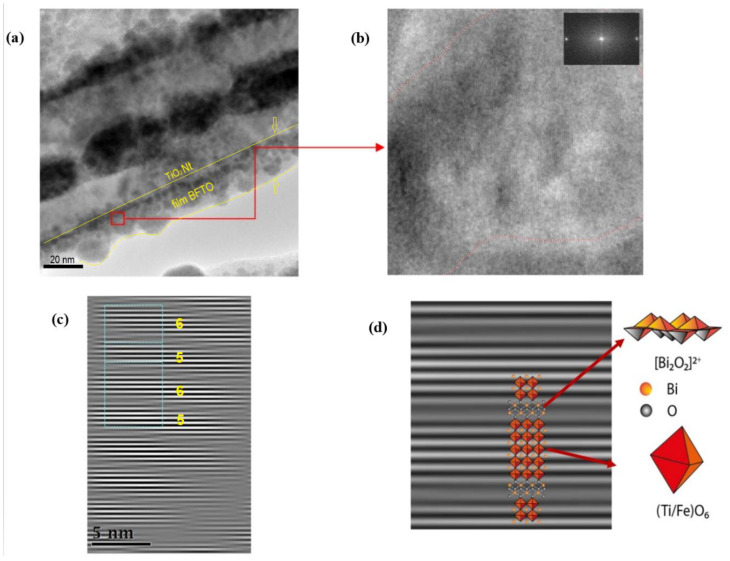
Transmission electron microscopy (TEM) analysis: (**a**) image of an ALD-coated single TiO_2_ Nt, (**b**) enlarged area (7 × 7 nm^2^) near the nanotube–film interface where the layered structure is visualized and (**c**,**d**) Fourier masked micrographs of the layered structure region.

As can be seen from Figure 2a, the substrate surface before ALD is coated with an array of vertically oriented TiO_2_ Nt with a diameter of about 100 nm and a length of about 2 µm. Figure 2b demonstrates the surface of nanotubes after ALD of Bi_2_O_3_-FeO_x_. It can be seen that the surface of the nanotubes is coated with an inhomogeneous layer of nanostructures with a diameter of about 100 nm. There are pores up to 50 nm wide through which the surface of the nanotubes is visible. TEM studies were carried out for a more detailed characterization of the coating (Figure 3). A TEM image of a single ALD-coated TiO_2_ Nt is shown in Figure 3a. It is shown that a single nanotube is uniformly covered with a film about 20 nm thick. The film contains nanocrystals with grain sizes of 10–15 nm (dark areas) together with the presence of an amorphous phase (light areas). Experimental data on the formation kinetics of the layered perovskite-like compounds in the BFTO system showed that the Aurivillius phase begins to form at 600 °C [21]. Figure 3b shows an enlarged area near the nanotube–film interface. It shown that a complex layered structure is formed after annealing at 660 °C in the nanotube–film interface, which is typical to the Aurivillius phases. The crystal lattice of all Aurivillius phases consists of perovskite-like layers (A_n-1_B_n_O_3n+1_)^2-^, which alternate with (Bi_2_O_2_)^2+^ bismuth-oxygen layers (Figure 3c). Fourier analysis of the region with a high resolution (Figure 3c,d) allowed to visualize one-dimensional (1D) phase-modulated structures consisting of two different layered Aurivillius phases with alternating the number of five and six perovskite-like layers (Figure 3c). Modulation of the interface in the periodically laid structures was previously reported; however, it was usually carried out by doping various positions of the structure with alkaline-earth or rare-earth metal ions [22,23]. The transition between layers with a different number of blocks is accompanied by stacking defects in the (Bi_2_O_2_)^2+^ layer. Such interfaces between 5- and 6-layer blocks can lead to complex deformations and distortions. The perovskite structure remains in this case and regular lattice distortions appear in certain regions without changing the general layout and forming regions with morphotropic phase transitions [24]. Such regions are probably associated with the substitution of Fe^3+^ ions in a narrow range of 0.58–0.65 for Ti^4+^ ions in octahedral cells [25]. Kikuchi [26] also explains the formation of such regions by the 6% difference in the ionic radii of iron and titanium, which leads to a shift of these ions relative to (Bi_2_O_2_)^2+^ layers.

**Figure 4:**
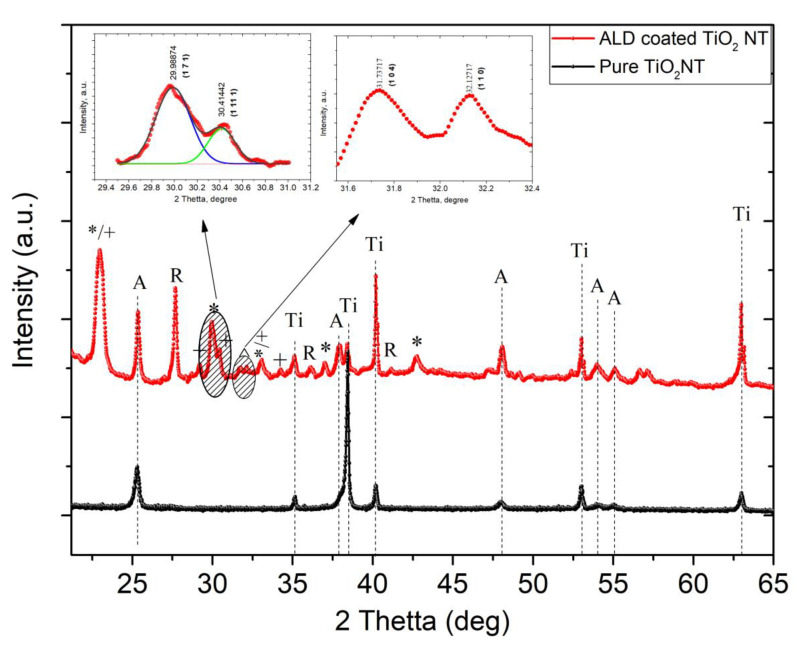
XRD patterns of pure and ALD-coated TiO_2_ Nt. A—anatase; Ti—titanium; R—rutile; *—Bi_4_Ti_3_O_12_; +—Bi_6_Fe_2_Ti_3_O_18_; ^—BiFeO_3_. Insert (**left**)—deconvoluted Bi_4_Ti_3_O_12_/Bi_6_Fe_2_Ti_3_O_18_ peaks, (**right**) —BiFeO_3_ peaks.

Typical XRD patterns of the pure and ALD-coated TiO_2_ Nt grown on Ti substrate are shown in Figure 4. It is revealed that the pure TiO_2_ Nt exhibit a phase of anatase (ICSD reference code: 98-015-4603) and Ti (ICSD reference code: 98-065-3275). The overlap of XRD peaks occurred because of large amount of phases in the obtained structure. After annealing at 660 °C, an initial phase of anatase partially transformed into the rutile phase with the characteristic diffraction peak at 2θ = 27.7° (110) [27]. The anatase/rutile ratio in ALD-coated TiO_2_ Nt calculated using Equation (3) amounted to 0.7.

(3) $$X_{A}=\frac{0.79\cdot I_{A}}{0.79\cdot I_{A}+I_{R}{}^{\prime }}$$

where I_A_ and I_R_ are the integrated intensities of the most intense peaks of anatase (011) and rutile (110) phases, respectively. The diffraction peaks observed at 2θ = 30.1° (171), 33.1° (200) and 42.8° (113) indexed to orthorhombic Bi_4_Ti_3_O_12_ with Aba2 space group (ICSD reference code: 98-008-7809) [28]. The peaks can be indexed to a pure hexagonal structure with R3c space group for BiFeO_3_ observed at around 2θ = 31.7° (104) and 32.1° (110) (ICSD Reference code: 98-001-5299) [29] and shown in the right insert in Figure 4. The other peaks correspond to orthorhombic Bi_6_Fe_2_Ti_3_O_18_ with Fmm2 space group. Deconvolution of the peaks in the left insert in Figure 4 indicates the phase of Bi_4_Ti_3_O_12_ at 29.9° (171) and of Bi_6_Fe_2_Ti_3_O_18_ at 30.4° (11 11). Presence of Bi_4_Ti_3_O_12_ and BiFeO_3_ phases confirms the suggested method of structure formation of Aurivillius phase.

**Figure 5:**
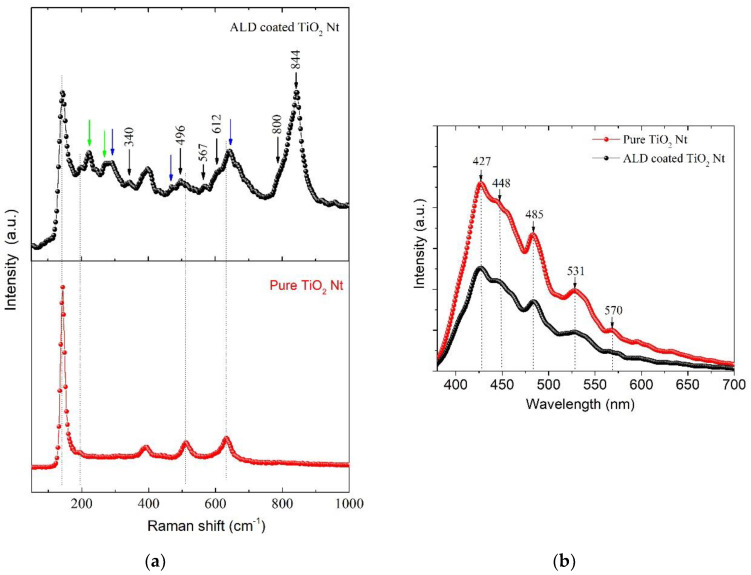
(**a**) Raman and (**b**) photoluminescence spectra for pure and ALD-coated TiO_2_ Nt.

Since Raman spectroscopy is sensitive to atomic displacements, it can provide valuable information on lattice properties and structural transitions. Raman spectra were obtained and the data are presented in Figure 5a. For comparison, the spectra of the pure TiO_2_ Nt crystallized in the anatase phase are also presented. The low-frequency region of the Raman spectra of layered bismuth compounds (below 200 cm^−1^) refers to vibrations of Bi^3+^ ions in the (Bi_2_O_2_)^2+^ layer and vibrations of Bi^3+^ ions relative to oxygen octahedra. The high-frequency modes above 200 cm^−1^ characterize the bending and tensile modes of the BO_6_ octahedron [30]. The characteristic TiO_2_ modes of anatase and rutile phases are observed in the spectra [31]. Rutile appears because of anatase–rutile transformation that occurs at 660 °C during annealing. It should be noted that almost all high-frequency modes are asymmetric; therefore, they can be interpreted as a superposition of several closely located Raman modes. The peak at 340 cm^−1^ corresponds to the torsional bending of the octahedra Ti/FeO_6_. Modes at 567 and 800 cm^−1^ are connected with stretching octahedral O-Ti/Fe chains between (Bi_2_O_2_)^2+^ layers. The asymmetric peak in the 844 cm^−1^ region corresponds to fully symmetric stretching vibrations of the O-Ti-O and O-Fe-O bonds in the Ti/FeO_6_ octahedra. The appearance of phonon modes at 567 and 706 cm^−1^ occurs due to a combination of the stretching of the Bi-Fe-O bond and the octahedral bending [32,33]. The Raman modes at about 222, 267 and 535 cm^−1^, are attributed to the internal vibrational modes of Ti/FeO_6_ octahedrons in BFTO [32]. In addition, the characteristic peaks of BiFeO_3_ (green arrows) and Bi_4_Ti_3_O_12_ (blue arrows) are observed in the spectrum [34,35].

Photoluminescence (PL) analysis is commonly used to analyze the recombination rate of photo-generated electron-hole of TiO_2_. Herein, we conduct PL measurement for pure and ALD-coated TiO_2_ Nt samples (Figure 5b). PL radiation intensity refers to the recombination of excited electrons and holes. Lowering the PL intensity indicates a decrease in the probability of recombination. It can be seen that the emission spectra are similar. When compared with pure TiO_2_, the intensity of the PL signal for ALD-coated TiO_2_ Nt is much lower.

This is due to the reduction in the radiative recombination process—that is, the lower the recombination, the weaker the PL signals are [36,37]. The spectra show broad emission in the visible range (350–600 nm). Two PL peaks at 427 and 448 nm correspond to self-trapped excitons (STE) localized on TiO_6_ octahedra and oxygen vacancies with two electrons, respectively (F centers) [38]. The broad peak at 531 nm is caused by an oxygen vacancy with one captured electron (F^+^ center) [39]. The peaks at 485 and 570 nm correspond to oxygen vacancies with one electron and surface oxygen vacancies (Vo). The presence of oxygen vacancies contributes to a more efficient photocatalytic process. The effective charge carrier separation extends the reactive electron and hole lifetimes [40] and enhances the photocatalytic activity of the photocatalysts. 

**Figure 6:**
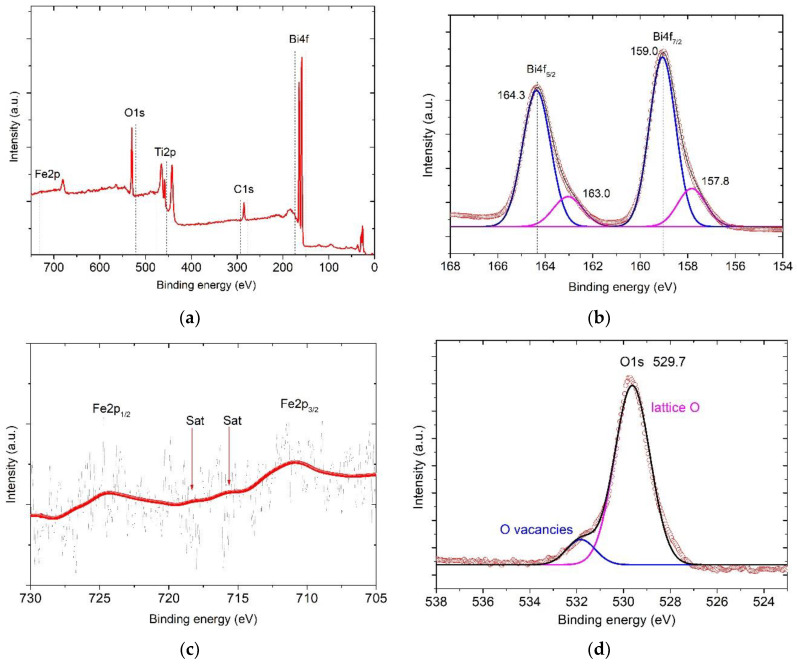

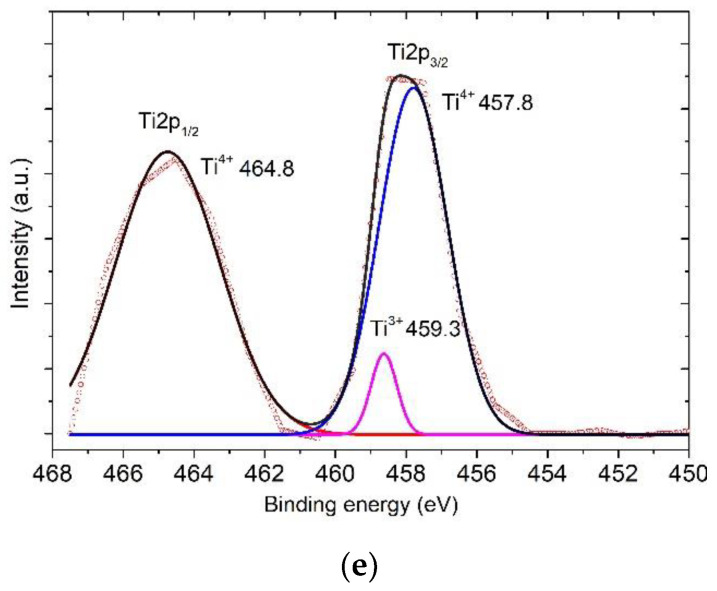
(**a**) Survey X-ray photoelectron spectrometer (XPS) spectra for the ALD-coated TiO_2_ NT. (**b**) High-resolution Bi 4f spectra; (**c**) high-resolution Fe 2p spectra; (**d**) high-resolution O 1s spectra; (**e**) high-resolution Ti 2p spectra.

To determine the valence state of ions for ALD-coated TiO_2_ NT, room temperature X-ray photoelectron spectroscopy studies were performed. Figure 6a shows the X-ray photoelectron spectroscopy survey spectra from 0 to 750 eV. The core level energies associated with Fe, Bi, Ti, O and C elements are identified. Five regions related with Fe 2p, Bi 4f, Ti 2p and O 1s of the survey XPS spectra for compounds were analyzed. High resolution spectra of Bi4f core level are shown in Figure 6a. Peaks Bi 4f_7/2_ and Bi 4f_5/2_ at ~159.0 eV and ~164.3 eV, respectively, correspond to Bi_2_O_3_. The energy of the spin-orbit splitting of the Bi 4f doublet is equal to 5.3 eV (the energy difference between Bi 4f_7/2_ and Bi 4f_5/2_) and consistent with previously reported experimental and theoretical values. Probably, the asymmetry of the Bi 4f_7/2_ and Bi 4f_5/2_ peaks indicates the presence of bismuth ions with other oxidation states that differ from +3 [41].

The spectra of Fe 2p core level are shown in Figure 6c. The signal is weak and a similar situation was observed in other works [42,43] but the reason is still unknown. The peaks’ asymmetry indicates the presence of mixed valence of iron cations [44].

Oxygen vacancies will occur in the presence of Fe^2+^ ions in the structure [45], which are necessary to maintain electroneutrality of the system, according to the Kröger–Vink notation:

(4) $$2{\mathrm{Fe}}^{3+}+1/2O_{o}↔2{\mathrm{Fe}}^{2+}+V_{\ddot{O}}$$

Oxygen vacancies reduce intrinsic defects on particle surfaces, which are a well-known capture center for carrier recombination. This effect promotes electron–hole separation in lattices and causes increasing photocatalytic efficiency [46].

The core-level spectra of Ti 2p are shown in Figure 6e. The spin-orbit splitting of Ti 2p_1/2_ and Ti 2p_3/2_ peaks located at around 464.7 and 457. 8 eV, respectively, corresponds to the binding energy of Ti 2p_1/2_ and Ti 2p_3/2_. These peaks are consistent with Ti^4+^ in TiO_2_ lattice [47]. Furthermore, the shoulder Ti 2p_1/2_ at binding energy 459.3 eV corresponds to Ti^3+^ in Ti_2_O_3_ [48]. The appearance of Ti^3+^ is caused by the partial replacing of titanium atoms by iron atoms in TiO_6_ octahedra. A similar effect was observed at doping of TiO_2_ films with iron ions [49]. In this case, at Ti^4+^/Ti^3+^ transition, oxygen vacancies will be formed in the structure for charge balance, similar to Fe^3+^/Fe^2+^ transition, as was mentioned above. It could be confirmed by high resolution XPS spectra of the O 1s peak (Figure 6d). The intensive peak at 529.7 eV belongs to lattice oxygen. The shoulder at 531.5 eV corresponds to defect oxygen components as oxygen vacancy.

**Figure 7:**
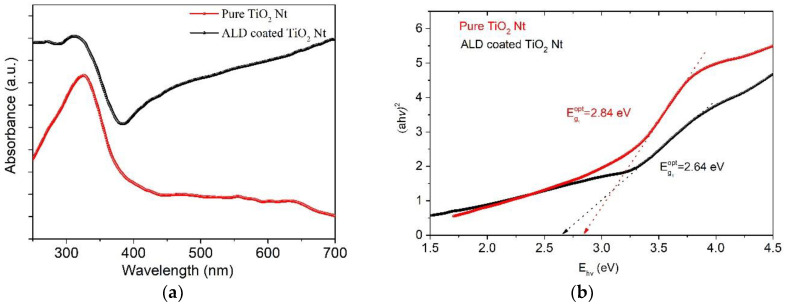

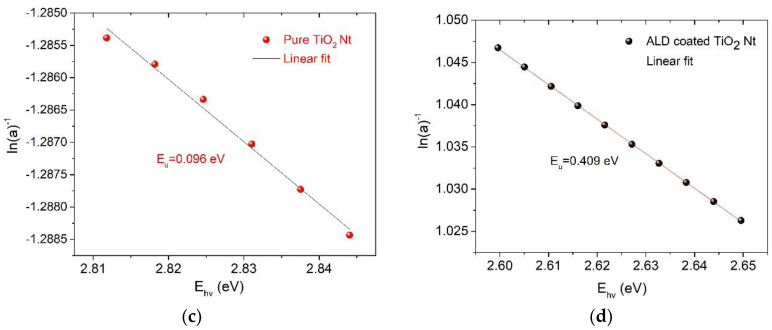
Optical properties of pure and ALD-coated TiO_2_ Nt. (**a**) UV-Vis spectra, (**b**) Tauc plots for indirect transition and (**c**) determination of Urbach energy for pure and (**d**) ALD-coated TiO_2_ Nt.

The optical properties of the samples were studied by UV-Vis diffuse reflection. Figure 7a shows the diffuse reflectance spectra presented in the Kubelka–Munk absorption coordinates. The appearance of the shoulder in the visible region and the shift of the absorption edge of the heterostructure towards large wavelengths could be observed. The absorption at the 330 nm wavelength is connected with the indirect transition of phonons from the edges to the center of the Brillouin zone [50]. It was shown by Sun et al. [9], by ab initio calculations, that the valence band in layered perovskites mainly consists of hybrid orbits O 2p + Fe t_2g_ + Bi 6s and the conduction band consists mainly of orbits Ti 3d + Fe e_g_. In this case, the electronic excitation from hybridized orbits O 2p + Fe t_2g_ + Bi 6s to Fe e_g_ orbits should be responsible for the absorption of photons of visible light, and the electronic excitation from hybrid orbits O 2p + Fe t_2g_ + Bi 6s to 3d orbits of Ti corresponds to the absorption of photons of ultraviolet light. It can be concluded that using UV-Vis radiation will be more effective for photocatalysis of ALD-coated TiO_2_ Nt. Figure 7b shows the energy (h*ν*) dependence of the direct allowed inter-band transition (αhν)^2^. The band gap of the films was estimated by the traditional Tauc’s relation:

(5) $$\alpha h\nu =A{\left(h\nu -E_{g}\right)}^{n}$$

where A—a constant; h*ν*—photon energy; α—absorption coefficient; E_g_—energy of the optical band gap; n—½ in the case of indirect and 2 in the case of direct inter-band transition. The direct and indirect band gaps for pure TiO_2_ Nt are 2.84 and 3.05 eV, for ALD-coated TiO_2_ Nt are 2.67 and 2.31 eV, respectively. As expected, the (E_g_) of the ALD-coated TiO_2_ Nt decreases due to the fact that the E_g_ of pure TiO_2_ Nt is higher than in compounds of the Aurivillius phases [9,51]. Here, the decrease in the value of the band gap is apparently explained by the presence of additional energy states near the edge of the conduction band, which is caused by the disorder of the crystal lattice formed during the deposition of the coating. It is known that the distribution of the density of electronic states determines the physical characteristics of a material and the processes occurring in it: optical absorption, conductivity and photoconductivity. The density of localized electronic states in synthesized systems can be evaluated from the Urbach characteristic energy, which can be calculated from the rate of exponential decay of the “tail” in the absorption spectrum. The width of the Urbach tail is an indicator of the disorder in the material. Urbach energy tail is evaluated according to the Urbach law [52].

(6) $$\alpha =\alpha_{0}\exp{\left(\frac{h\nu }{E_{U}}\right)}^{}$$

(7) $$E_{U}={\left[\frac{\mathrm{dln}\alpha }{\mathrm{dh}\nu }\right]}^{-1}$$

where α_0_—constant; E_U_—Urbach energy, which characterizes the slope of the exponential limit. The E_U_ is calculated from the inverse dependence of the slope ln α on hν in the region slightly below the band gap [53]. Results are presented in Figure 7c,d. The calculated values of Urbach energy are 96 meV for pure TiO_2_ Nt and 409 meV for ALD-coated TiO_2_ Nt. A significant increase in the E_U_ for ALD-coated TiO_2_ Nt indicates the appearance of impurity energy levels in the band gap. These energy levels can be associated with the coexistence of one-dimensional (1D) phase-modulated structures consisting of two different layered Aurivillius phases with alternating the number of five and six perovskite-like layers with different Fe/Ti ratio in the octahedra and structural disorder due to the presence of oxygen and bismuth vacancies [54]. This is consistent with the conclusion of B. Choudhury and A. Choudhury [55] where they mention that the energy increase is caused by the presence of oxygen vacancies and structural disorder at the interface of different phases. It confirms that the PL and UV-Vis spectroscopy data are in good agreement and indicate the prospect of using the obtained materials as photocatalysts, since the oxygen vacancies play an essential role in photocatalytic reactions [56].

**Figure 8:**
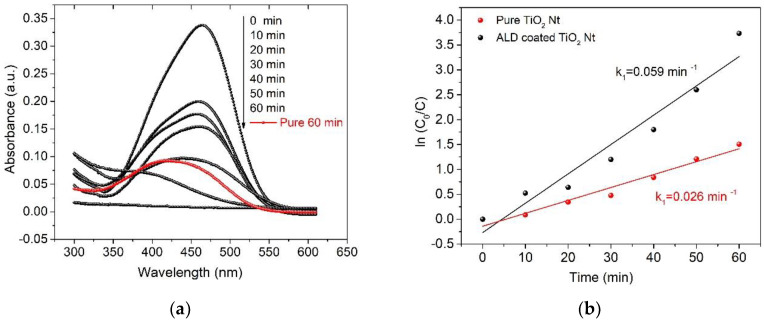

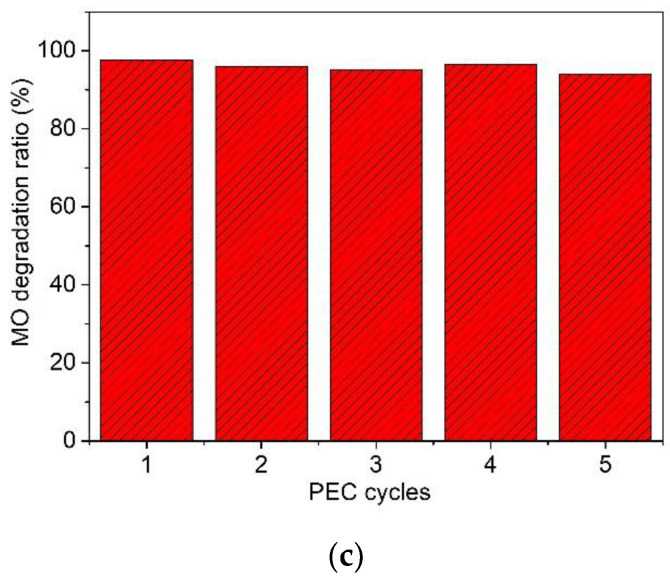
Temporal absorption spectral patterns of methyl orange (MO) during the photoelectrocatalytic process (**a**) using pure and ALD-coated TiO_2_ Nt under UV-Vis light irradiation. (**b**) First-order reaction kinetics curves. (**c**) Photoelectrocatalysis cycles of MO degradation using ALD-coated TiO_2_ Nt under UV-Vis light irradiation.

The degradation of MO solution with 5 mg/L concentration was utilized as a probe to investigate the photoelectrocatalytic activities of both pure and ALD-coated TiO_2_ Nt for comparison. Figure 8a shows the region of the absorption spectrum of the MO solution around the wavelength of 466 nm. This sector is characterized by the presence of -N=N- bonds, of which the dynamics reflect the discoloration process. The red line (Figure 8b) shows the residual MO concentration after 60 min of the PEC process using pure TiO_2_ Nt as the photoelectrode. The black lines (Figure 8a) show the kinetics of the PEC process using ALD-coated TiO_2_ Nt as the photoelectrode. It can be seen that the sample of ALD-coated TiO_2_ Nt showed the best PEC activity. Almost 100% of MO removal was observed in 60 min of the process, while for pure TiO_2_ Nt, it was 72%. PEC degradation of MO with booth photoelectrodes obeys the first-order reaction kinetics as shown in Figure 8b. The decomposition rate of MO using ALD-coated TiO_2_ Nt increases more than two times according to the calculated values of the rate constants. A cycling test was carried out since the stability of the photoelectrodes is very important. Figure 8c presents the long-term stability of ALD-coated TiO_2_ Nt in the PEC MO degradation process. As can be seen after five cycles, the PEC degradation ratio is maintained at 95%, suggesting good stability.

**Figure 9:**
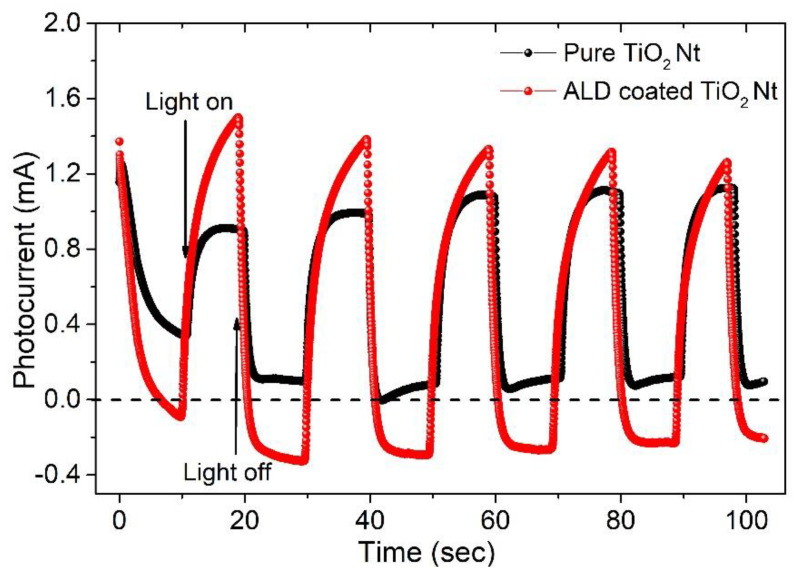
Transient photocurrent responses under Ultraviolet–Visible (UV-Vis) light irradiation.

The enhancement of the photon excitation efficiency of electrons due to the ALD coating is confirmed by the observation of the difference in photocurrent between pure and ALD-coated TiO_2_ Nt. The photocurrent was measured under pulsed irradiation of UV-Vis light. The induced photocurrents of the samples are well correlated with pulsed irradiation of UV-Vis light (Figure 9). The ALD-coated TiO_2_ Nt have a stronger photoresponse than the pure TiO_2_ Nt. The photocurrent peak of ALD-coated TiO_2_ Nt is about 23% higher than for pure nanotubes because of easier photoinduced electronic excitation. Both photoelectrodes showed relatively good reproducibility and stability when lights were turned on and off. Both samples showed the anode photocurrent when the light was turned on, which is typical for n-type semiconductors. However, when the illumination is turned off, a dark negative current is detected in the cell with the ALD-coated TiO_2_ Nt and this value slowly reached saturation and stabilized. A similar effect was observed by B. Ouyang et al. [57], where the authors described the SnS/ZnO system with a p-n heterojunction and explained the appearance of a negative current after the lighting is turned off by the complex effect of photoexcitation arising from photoabsorption and thermo-potential inside SnS. In view of the presence of various properties (piezoelectric effect) in layered compounds of the Aurivillius phases, which can be responsible for this effect, the explanation in our case is still not clear. Further studies are needed in order to understand the observed phenomenon.

This result can be explained considering that TiO_2_ is an n-type semiconductor [58] and layered perovskites of the Aurivillius phase exhibit ionic-p-type electronic mixed conduction [59]. The p-type electrical conductivity is due to oxygen vacancies in the bismuth oxide layer. Thus, a p-n heterojunction is formed at the BFTO–TiO_2_ Nt interface, in which an internal electric field is present because of the spatial separation of charges. According to literature [8], the edge of the n-type TiO_2_ conduction band (CB) is located above the CB of the BFTO edge, which makes the transitions of the photogenerated electrons from BFTO to TiO_2_ thermodynamically unfavorable; however, holes can easily transfer from n-type TiO_2_ to p-type BFTO [60]. This limits the recombination and contributes to a more efficient separation of photogenerated electron–hole pairs. 

## 4. Conclusions

One-dimensional phase-modulated structures of layered Aurivillius phases with alternating 5 and 6-layer perovskite-like blocks were obtained on the surface of TiO_2_ Nt by ALD using the methodology of a two-layer Bi_2_O_3_-FeO_x_ sandwich structure. It is shown that the layered structure is formed due to self-organization during annealing at 660 °C. The results of PL spectroscopy indicate a high application potential in photocatalysis and photoelectrochemistry due to a decrease in the radiative recombination of charges. Diffuse reflection spectroscopy demonstrated a narrowing of the band gap due to defective energy states associated with disorder of the crystal lattice. The 4.2-times increase in the Urbach energy for ALD-coated TiO_2_ Nt characterizes the structural disorder associated with the presence of oxygen and bismuth vacancies and coexistence of 1D phase-modulated structures consisting of two different layered Aurivillius phases with alternating the number of five and six perovskite-like layers with different Fe/Ti ratio in the octahedra. Transient photocurrent responses under UV-Vis light irradiation show that the ALD coating benefits the efficiency of photon excitation of electrons. The results of the PEC experiments and MO photodegradation demonstrate the significant potential of the synthesized structure as a photocatalyst.

## Supplementary Materials

The following are available online at https://www.mdpi.com/2079-4991/10/11/2183/s1, Figure S1: Emission spectrum of 250 W discharge mercury vapor lamp with a preliminary removed phosphor layer.
