# Supplementary material for: Atomic Layer Deposition of Mixed-Layered Aurivillius Phase on TiO2 Nanotubes: Synthesis, Characterization and Photoelectrocatalytic Properties
Source: Nanomaterials (Basel). 2020 Nov 2;10(11):2183. doi: 10.3390/nano10112183 (PMC7693954; doi:10.3390/nano10112183)
Supplement: Supplementary file 1 [file nanomaterials-10-02183-s001.pdf]

nanomaterials

Supplementary Materials

Atomic Layer Deposition of Mixed-Layered Aurivillius Phase on TiO_2 Nanotubes: Synthesis, Characterization and Photoelectrocatalytic Properties

Farid Orudzhev [1*], Shikhgasan Ramazanov [1], Dinara Sobola [1,2,3], Abdulgalim Isaev [1], Chuanyi Wang [4], Asiyat Magomedova[1], Makhmud Kadiev[1], Kasinatan Kaviyarasu[5,6]

1 Dagestan State University, st. M. Gadjieva 43-a, Dagestan Republic, Makhachkala 367015, Russia
2 Department of Physics, Faculty of Electrical Engineering and Communication, Brno University of Technology, Technická 2848/8, Brno 61600, Czech Republic Laboratory of Environmental Science and Technology
3 Central European Institute of Technology BUT, Purkyňova 123, Brno 61200, Czech Republic
4 Shaanxi University of Science & Technology, Xi'an, Shaanxi 710021, China
5 UNESCO-UNISA Africa Chair in Nanoscience's/Nanotechnology Laboratories, College of Graduate Studies, University of South Africa (UNISA), Muckleneuk Ridge, P O Box 392, Pretoria, South Africa.
6 Nanosciences African Network (NANOAFNET), Materials Research Group (MRG), iThemba LABS-National Research Foundation (NRF), 1 Old Faure Road, 7129, P O Box 722, Somerset West, Western Cape Province, South Africa.
* Correspondence: ff.orudzhev@dgu.ru; Tel.: +7-928-866-26-22

Photoelectrochemical measurement

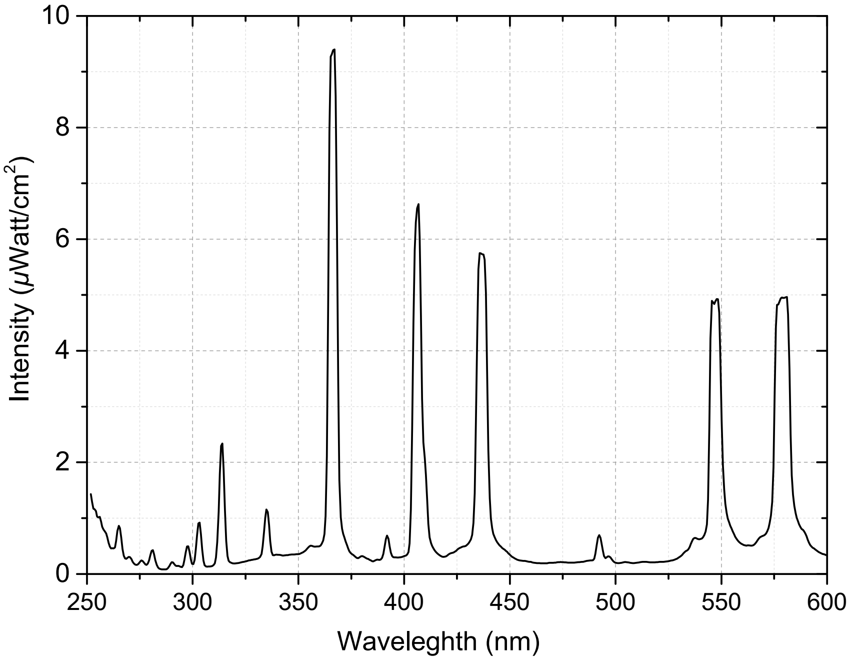

Figure S1. Emission spectrum of 250 W discharge mercury vapor lamp with a preliminary removed phosphor layer.
